# Bunyaviral N Proteins Localize at RNA Processing Bodies and Stress Granules: The Enigma of Cytoplasmic Sources of Capped RNA for Cap Snatching

**DOI:** 10.3390/v14081679

**Published:** 2022-07-29

**Authors:** Min Xu, Magdalena Mazur, Nigel Gulickx, Hao Hong, Hein Overmars, Xiaorong Tao, Richard Kormelink

**Affiliations:** 1 Laboratory of Virology, Department of Plant Sciences, Wageningen University & Research, Droevendaalsesteeg 1, 6708 PB Wageningen, The Netherlands; min.xu1011@gmail.com (M.X.); malena.mazur@gmail.com (M.M.); nigel.gulickx@gmail.com (N.G.); 2 Key Laboratory of Integrated Management of Crop Diseases and Pests, Ministry of Education, Department of Plant Pathology, Nanjing Agricultural University, Nanjing 210095, China; honghao_ahjx@163.com (H.H.); taoxiaorong@njau.edu.cn (X.T.); 3 Laboratory of Nematology, Department of Plant Sciences, Wageningen University & Research, Droevendaalsesteeg 1, 6708 PB Wageningen, The Netherlands; hein.overmars@wur.nl

**Keywords:** bunyavirus, N protein, cap snatching, processing body, stress granule, cytoplasmic RNA spot

## Abstract

Most cytoplasmic-replicating negative-strand RNA viruses (NSVs) initiate genome transcription by cap snatching. The source of host mRNAs from which the cytoplasmic NSVs snatch capped-RNA leader sequences has remained elusive. Earlier reports have pointed towards cytoplasmic-RNA processing bodies (P body, PB), although several questions have remained unsolved. Here, the nucleocapsid (N) protein of plant- and animal-infecting members of the order *Bunyavirales*, in casu Tomato spotted wilt virus (TSWV), Rice stripe virus (RSV), Sin nombre virus (SNV), Crimean-Congo hemorrhagic fever virus (CCHFV) and Schmallenberg virus (SBV) have been expressed and localized in cells of their respective plant and animal hosts. All N proteins localized to PBs as well as stress granules (SGs), but extensively to docking stages of PB and SG. TSWV and RSV N proteins also co-localized with Ran GTPase-activating protein 2 (RanGAP2), a nucleo-cytoplasmic shuttling factor, in the perinuclear region, and partly in the nucleus when co-expressed with its WPP domain containing a nuclear-localization signal. Upon silencing of PB and SG components individually or concomitantly, replication levels of a TSWV minireplicon, as measured by the expression of a GFP reporter gene, ranged from a 30% reduction to a four-fold increase. Upon the silencing of RanGAP homologs *in planta*, replication of the TSWV minireplicon was reduced by 75%. During in vivo cap-donor competition experiments, TSWV used transcripts destined to PB and SG, but also functional transcripts engaged in translation. Altogether, the results implicate a more complex situation in which, besides PB, additional cytoplasmic sources are used during transcription/cap snatching of cytoplasmic-replicating and segmented NSVs.

## 1. Introduction

The RNA-dependent RNA polymerase (RdRp) from most segmented, negative-strand (-) RNA viruses lacks a methyltransferase activity that normally is needed to provide a 5′terminal m^7^G cap on viral RNA transcripts to support their translation [1]. To circumvent this problem, these viruses employ cap snatching, a process during which capped-RNA leader sequences are cleaved from host cellular messengers and used to align on the 3′ end of viral RNA segments to prime transcription. In recent years, 3D folding structures have been resolved of the viral RdRp complex from the influenza virus and several other viruses that employ cap snatching, which has revealed some structural similarities and supports a highly conserved mechanism of genome transcription initiation [2,3]. For the nuclear replicating influenza virus, the RdRp complex consists of three subunits called PA, PB1 and PB2. Although PA contains the endonuclease domain [4] and PB1 contains the catalytic core domain of the RNA polymerase, PB2 contains the cap-binding domain [5,6]. In contrast, for all cytoplasmic replicating segmented (-)RNA viruses employing cap snatching, i.e., members of the order *Bunyavirales*, and from here onwards referred to as bunyaviruses, the RdRp consists of a single protein that holds an endonuclease domain in the N terminus and six polymerase motifs, characteristic of the RdRp of (-)RNA viruses, in the central region [7,8]. However, the rest of the large RdRp proteins, ranging in size from ~240 kDa to ~460 kDa between these members, are so far functionally uncharacterized, partly due to the absence of sequence homology with other proteins. A cap-binding domain (CBD), according to alignments with the influenza virus polymerase units, is suspected to map to the C-terminal domain of their RdRps, has not been found [7]. Despite an overall structural homology more recently found between the CBD of influenza polymerase and the C-terminal domain of the RdRp from those of LaCrosse virus (LACV), Rift valley fever virus (RVFV) and the California Academy of Sciences Virus (CASV) [9,10,11], their low affinity for cap structures does not explain how they are able to compete with high-affinity cellular cap-binding proteins such as eIF4E, which strongly sequesters cytoplasmic mRNAs and thereby prevents access to their capped-RNA leader sequences for the bunyaviral transcriptase complex. 

Furthermore, a C-terminal part of the *Reptarenavirus* (*Bunyavirales*; *Arenaviridae*) polymerase, with a structural resemblance to the cap-binding domain of the influenza virus, presented a degenerate cap-binding domain due to the absence of a typical structural sandwich of two aromatic residues, and failure to biochemically detect cap-binding [12]. 

Although these findings do not entirely rule out the possibility for the RdRp proteins of these bunyaviruses to harbor a CBD, they do raise the question whether or not another viral protein is also needed for transcription-replication of these viruses, e.g., whether the nucleocapsid (N) protein could harbor such a domain and/or possibly act in concert with RdRp to fulfil cap snatching. 

In the past two decades, studies have demonstrated that (multiple) base complementarity of the 3′-end residues of capped-RNA leader molecules to the viral RNA genome template promotes their usage as primers during viral genome transcription initiation. This feature has been observed with several plant- and animal-infecting bunyaviruses [13,14,15,16,17,18,19,20,21] and supports the idea that the mechanistic model for cap snatching is likely generic for all of these viruses. From all segmented (-)ssRNA viruses that employ cap snatching, the influenza virus is the only one that replicates in the nucleus where its viral RNA polymerase complex interacts with the large subunit of RNA Polymerase II (Pol II) via its C-terminal domain [22,23]. Being confined to RNA Pol II transcription sites, the influenza virus has direct access to (a continuous supply of) cellular capped pre-mRNAs from which capped-RNA leaders are snatched to initiate viral genome transcription. However, where and how the bunyaviruses snatch-capped-RNA leaders still remains somewhat elusive, as also reviewed by Olschewski et al. (2020) [24]. It is tempting to assume that bunyaviruses similarly use a cost-effective strategy for cap snatching and are confined to specific areas in the cytoplasm enriched with mRNA as source for capped-RNA leaders [25]. Candidates for these are two major cytoplasmic RNA granules, i.e., RNA processing bodies (P body, PBs) and stress granules (SGs) [26]. P bodies are constantly present in the cell and play a major role in cellular RNA turnover as they contain the RNA decay machinery (waste bin) of the cell. SGs are dynamic and transient foci enriched in (functional) mRNAs stalled in their translation when there is stress. P body and SG are spatially, functionally and dynamically linked [27]. P body and SG are also frequently found to cooperate during viral infection in both animals and plants, during which they may interact with viral proteins and exhibit pro- or antiviral activities [28,29,30,31,32]. 

Earlier observations made on the Sin Nombre (SNV) hantavirus pointed towards P bodies as the source for capped-RNA leaders. Not only its nucleocapsid (N) protein co-localizes with the P body marker protein mRNA Decapping enzyme 1 (Dcp1) but the virus also seems to preferably snatch cap leader sequences from transcripts that target to P bodies via the nonsense-mediated decay (NMD) pathway, a cellular surveillance mechanism that detects mRNA transcripts containing a premature termination codon (PTC) [19,33]. However, rates of cap snatching/viral transcription for SNV using PTC containing transcripts are similar in normal and P-body-deficient cells and argues against P bodies being the major source for capped-RNA leader molecules [19,33]. This raises the question whether P bodies present the (first and sole?) source for capped RNA leaders to support cap snatching, as initially postulated by Mir et al. [33], and whether or not other cytoplasmic RNA granules or foci present an (additional) source for capped-RNA leaders. SGs, due to their intimate relation with PBs, could present a candidate for this.

Considering the highly conserved nature of cap snatching and the failure so far to proof a fully functional and primary cap-binding domain in the viral RNA polymerase from all bunyaviruses, this study embarked from a concept in which the N protein is postulated to play a role in the recognition of host mRNAs by (in)directly mediating the binding to 5′ cap-structures. This idea is supported by the observations that (1) the SNV hantavirus N protein shows affinity to the 5′ cap of mRNAs and plays an important role in the initial stages of translation initiation [33,34,35], (2) the Junin (JUNV), Tacaribe (TCRV) and Pichinde (PICV) arenavirus N proteins are able to interact with a 7 methyl-guanosine (cap) [36] and (3) the Crimean-congo hemorrhagic fever (CCHFV) nairovirus N protein and TSWV tospovirus N protein enhance translation of viral mRNAs [37,38,39]. If this is true, the (indirect) affinity to 5′ cap structures will direct and concentrate these N proteins at cytoplasmic foci enriched for capped RNA, as previously observed with SNV [33]. In light of all these findings a recent study on TSWV has also shown its N protein to (partially) co-localize with P bodies and that the P body decapping machinery affects TSWV accumulation. When taking out a P body decapping element, TSWV accumulated to higher levels, but when plants were subjected to heat shock treatment, the higher accumulation was not observed [40]. However, whether P bodies function as a cap donor source for TSWV was not investigated in that study. In addition, the influence of heat treatment in this process was observed, but the authors did not further investigate heat-related SG formation nor its possible role as source for TSWV cap snatching. 

In this study the N proteins from several plant- and animal-infecting bunyaviruses have been localized relative to the major cytoplasmic RNA granules, and the involvement of those in viral replication was analyzed. Irrespective of their origin from a plant- or animal-infecting virus, expression in plant- and animal cells consistently revealed these N proteins to localize with P bodies and SG. Other than these RNA granules, the plant virus N proteins also localized to the perinuclear region, partly overlapping and interacting with RanGAP, an important nucleocytoplasmic transport factor.

## 2. Materials and Methods

### 2.1. Cloning of the Constructs 

All molecular standard techniques were performed using protocols as described (Sambrook and Russell, 2001). Primers used in the study are listed in the Supplementary Table S1 and were synthetized by Integrated DNA Technologies. Constructs made and used in this study were verified by sequence analysis (Eurofins Genomics, Ebersberg, Germany).

For the generation of fluorophore-fusion constructs, first eCFP, eGFP, eYFP and mRFP coding sequences were amplified using primers containing SacII restriction sites and subsequently cloned into SacII pre-digested pcDNA-DEST40 (Thermo Fischer Scientific, Breda, The Netherlands), resulting in pcDNA-DEST40-eCFP, pcDNA-DEST40-eGFP, pcDNA-DEST40-eYFP and pcDNA-DEST40-mRFP. 

TSWV orthotospovirus N and Rice stripe tenuivirus (RSV) N sequences were cloned from infected plant material. Crimean-Congo hemorhagic fever nairovirus (CCHFV) N, Sin nombre hantavirus (SNV) N, were kindly provided by drs. J. Barr and A. Panganiban, respectively. N gene sequences were re-amplified with primers containing attB1 and attB2 recombination sites and recombined into Gateway vector pDONR207 (Thermo Fischer Scientific, Breda, The Netherlands) using BP Clonase II (Thermo Fischer Scientific, Breda, The Netherlands). To obtain CMV driven fluorophore-tagged constructs for expression in mammalian cells, N gene constructs were transferred from the entry clones into either pcDNA-DEST40-eCFP, pcDNA-DEST40-eGFP, pcDNA-DEST40-eYFP or pcDNA-DEST40-mRFP by using Gateway LR clonase II (Thermo Fischer Scientific, Breda, The Netherlands). For expression in plant tissues, pDONR207 entry constructs harboring the plant virus N genes of TSWV and RSV were recombined into destination vector pK2GW7 by LR Clonase Enzyme Mix [41]. Mammalian RNA granules markers Hs decapping 1(HsDCP1), HsCaprin-1 and HsG3BP1 were kindly provided by Dr. N. Kedersha. Plant P body marker RFP-DCP1 was kindly provided by Dr. A. Maizel. Tandem zinc finger protein 1 fused with RFP (TZF1-RFP) and G3BP1-RFP were kindly provided by Dr. Björn Krenz. 

### 2.2. pEAQ-GFP (Modified), pEAQ-nsGFP and pEAQ-L-GFP Construction

GFP fragment was amplified and cloned into pEAQ-HT vector [42]. Site-directed mutagenesis was performed within the UTR leader sequence at nucleotide position 12. Primers with mutagenesis were used to amplify the whole plasmid followed by DpnI (NEB) enzymatic digestion to digest input plasmid DNA. The PCR fragment was purified, re-ligated and recovered as plasmid after transformation into bacteria. The pEAQ-GFP construct was modified to additionally introduce a premature stop codon at position nt10 of the GFP transcript leader sequence. To generate a long (L)-GFP construct, the GFP sequence was fused with a 2A sequence and two tandem LacZ sequences into pEAQ vector. 

### 2.3. Cell Culture, Transfection and Virus Infection

African green monkey kidney Vero E6 cells (ATCC CRI-1586) were cultured at 37 °C with 5% CO_2_ in Dulbecco’s Modified Eagle Medium (DMEM, Gibco, Waltham, MA, USA) supplemented with 10% fetal bovine serum (FBS, Fisher Inv., Camas, WA, USA) and penicillin and streptomycin (Sigma-Aldrich, St. Louis, MO, USA) at the final concentration of 100 U/mL and 100 µg/mL, respectively. Cells grown in a pre-seeded Nunc™ Lab-Tek™ II 8-Chamber Slide™ were transfected with 250 ng DNA (per construct) by using TransIT^®^-LT1 Transfection Reagent (Mirus Bio, Madison, WI, USA) in Opti-MEM serum (Gibco) following the manufacturer’s protocols. Schmallenberg orthobunyavirus (SBV) infection of animal cells was performed at a MOI of 2.2. Two hours post-virus infection, the cells were washed with PBS (Gibco) and the medium was replaced with fresh DMEM. 

### 2.4. Immunostaining

Cells were fixed for 10 min in 4% paraformaldehyde in PBS, followed by permeabilization for 10 min at RT in 0.1% sodium dodecyl sulphate (SDS) in PBS. After a PBS wash for three times, cells were blocked by incubation in 5% FBS for 30 min. To immunolocalize SBV N, primary monoclonal mouse antibody against SBV-N (1:100, kindly provided by Dr. K. Wernike), was used in combination with secondary antibody goat anti-mouse-Alexa Fluor 488 (1:1000; Thermo Fisher Scientific, Breda, The Netherlands) or goat anti-mouse-Alexa Fluor 633 (1:1000; Thermo Fisher Scientific, Breda, The Netherlands). In order to induce SGs, cells were treated with 0.5 mM sodium arsenite for 1 h prior to the immunostaining experiment and washed three times with PBS. The primary antibodies against eIF3 (polyclonal goat, 1:500; Santa Cruz Biotechnology, Dallas, TX, USA) were used in combination with secondary antibody donkey anti-goat-Qdot565 (1:50; Thermo Fisher Scientific) or donkey anti-goat-Alexa 568 (1:2000; Thermo Fisher Scientific, Breda, The Netherlands). Proteins were visualized in mammalian cells 24 h post transfection or post infection, unless stated otherwise. 

### 2.5. Plant Material, Transient Expression and Virus Inoculation

*Nicotiana benthamiana* were kept under greenhouse conditions (24 °C, during a regime of 16 h light/8 h dark per day). Six- to eight-week-old plants of *N. benthamiana* were used for all transient expression analyses and virus inoculations. 

N gene constructs and granule markers were transformed into *Agrobacterium tumefaciens* GV3101. Cells harboring different constructs were grown overnight at 28 °C. After centrifugation, the pellet was resuspended and treated with infiltration buffer (10 mM MgCl_2_, 10 mM MES, pH 5.9, and 150 μM acetosyringone) for 3 h at room temperature. *N. benthamiana* leaves were infiltrated with combinations of suspensions containing a final optical density at 600 nm (OD_600_) of 0.5 per construct. TSWV infection of plants was established by inoculation of virus from source inoculum using inoculation buffer (0.01 M Na_2_HPO_4_.H_2_O, 0.01 M Na_2_H_2_PO_4_.2H_2_O, pH7.5), 5 days before infiltration with marker gene constructs. In order to induce SGs, plants were subjected to a heat shock. To this end, plants were incubated at 37 °C for 45 min followed by 10 min under dark condition at room temperature. 

For virus-induced gene silencing (VIGS), *N. benthamiana* plants were infiltrated with suspensions of *A. tumefaciens* containing an infectious Tobacco Rattle virus (TRV) clone with sequences from the host gene to be targeted [43]. During VIGS silencing of host genes, a TRV-*GUS* gene construct was used as a negative control and TRV-*phytoene desaturase* (TRV-*PDS*) as positive control. Approximately 3 weeks later, when plants subjected to TRV-*PDS* exhibited chlorophyll bleaching in the top leaves, plants were superimposed with a TSWV infection by mechanical inoculation with virus inoculum, or by agroinfiltration with the TSWV replicon system. In brief, a codon-optimized TSWV RdRp construct, for usage in *N. benthamiana* and from which potential intron-splicing sites were removed, was cloned in a binary, 35S-driven expression vector and agroinfiltrated into *N. benthamiana* leaves together with binary expression constructs coding for the L, M and S-eGFP RNAs as described [44]. Prior to cloning of capped RNA leader sequences from viral mRNAs, SG formation was induced in plants by a heat treatment for 8 h at 37 °C. 

### 2.6. Confocal Microscopy and Green Fluorescence Microscopy Observation

Microscopical analysis was performed under Zeiss (Jena, Germany) LSM 510-META confocal laser scanning microscope with a ×60, 1.3-numerical aperture, oil-corrected objective and with the pinhole kept at 1 Airy unit throughout all experiments. For mRFP, excitation and emission 543 nm and 560–615 nm were used, respectively. For CFP this was 405 nm and 418–480 nm, respectively. Images were processed using a Zeiss 2010 CLSM and Image J viewer.

Samples infected by agroinfiltration of TSWV genome replicons were harvest at 60–72 h post infiltration and were analyzed by Olympus fluorescence microscopy. GFP excitation was performed at 488 nm and emissions captured at 500–530 nm. 

### 2.7. Quantification and Statistical Analysis

To quantify the number and size of the granules, ImageJ Analyze Particle Analyzer was used. To correct for background noise, a lower cut off for the size of the granules of 0.04 µm was applied. As close proximity of granules might result in a creation of one larger granule, an upper cut off of 6 µm was applied. For statistical analyses, at least 10 cells were collected for each treatment. Statistical analyses were performed by which means were compared by one-way ANOVA with Tukey’s post hoc test (*p*-value of 0.05). In cases when the tested data sets did not fulfil the assumptions of ANOVA, the Kruskal–Wallis test was performed with a *p*-value of 0.05. * *p*-value < 0.05, ** *p*-value ≤ 0.01, *** *p*-value ≤ 0.001. 

### 2.8. RNA Isolation and cDNA Synthesis

Total RNA was isolated using TRIzol reagent protocol (Invitrogen, Waltham, MA, USA). The RNA was treated with TURBO DNase for 30 min at 37 °C (TURBO DNA-free™ Kit, Invitrogen) followed by a DNase inactivation step. First-strand cDNA synthesis was performed on 500 ng total RNA per reaction using RT M-MLV (Promega, Madison, WI, USA) and random hexamers following the manufacturer’s protocol. Total RNA was isolated from plant materials using the same protocol. 

### 2.9. Quantitative Real-Time PCR (qRT-PCR)

After reverse transcription, first-strand cDNA was diluted five times prior to further analyses. Primers to genes were designed for quantitative analyses of RNA expression levels. The qRT-PCR was performed in an ABI 7500 Real-Time PCR system (Life Technologies, Carlsbad, CA, USA). *Actin 2* and *EF1a* served as internal controls to normalize the RNA levels of target gene expression between samples, using a relative quantification method.

## 3. Results

### 3.1. Viral N Proteins from Plant- and Animal-Infecting Members of the Bunyavirales Localize at PBs and SGs

**Figure 1:**
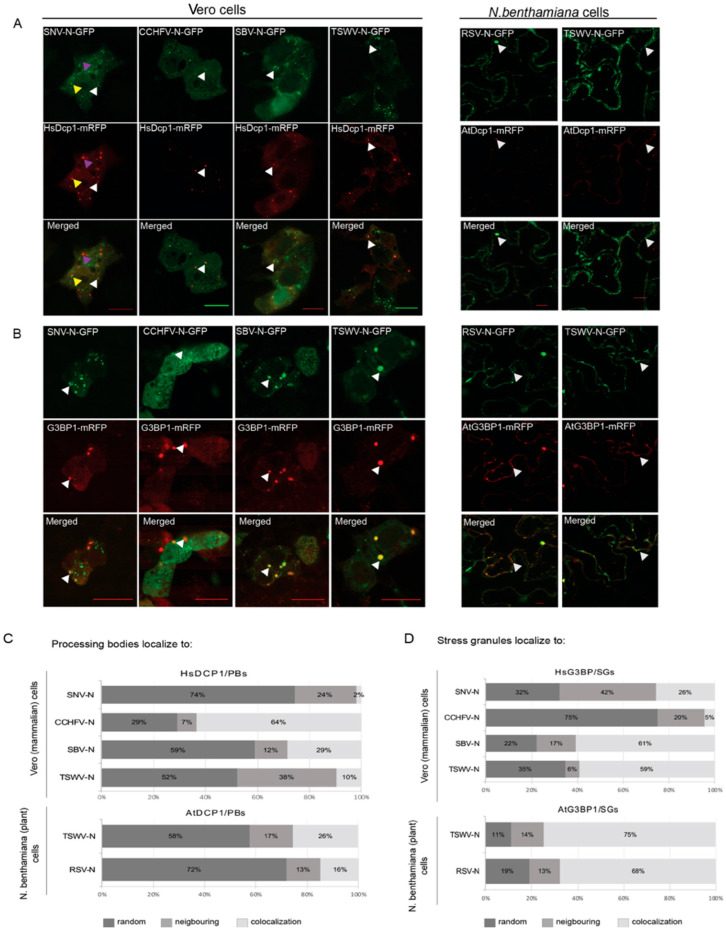
Localization and quantification of different NSV N proteins relative to PB/SG in plant cells and animal cells. (**A**) Fluorescence microscopy images of SNV, CCHFV, SBV and TSWV N-GFP protein localization relative to P body marker protein HsDcp1-mRFP (left panel) in Vero cells, and of RSV and TSWV N-GFP protein localization relative to P body marker protein AtDcp1-mRFP in plant cells (right panel). (**B**) Fluorescence microscopy images of SNV, CCHFV, SBV and TSWV N-GFP protein localization relative to SG marker protein HsG3BP1-mRFP (left panel) in Vero cells (subjected to arsenite treatment for SG-induction), and of RSV and TSWV N-GFP protein localization relative to SG marker protein AtG3BP1-mRFP in plant cells (subjected to a heat shock for SG-induction). (**C**) Quantification of P body localization relative to different N proteins. Colocalization, neighboring and random localization ratio were quantified for SNV, CCHFV, SBV and TSWV N protein in animal cells, and TSWV and RSV N protein in plant cells. (**D**) Quantification of SG localization relative to different N proteins. Colocalization, neighboring and random localization ratio were quantified for SNV, CCHFV, SBV and TSWV N protein in animal cells, and TSWV and RSV N protein in plant cells, after cells were treated for SG-induction. Bar is 10 μm.

To analyze whether a co-localization with P bodies is generic to the N protein from members of the order *Bunyavirales*, N proteins from several plant- and animal-infecting bunyaviruses were fused with GFP and transiently co-expressed with the cytoplasmic PB marker protein DCP1a fused to RFP in plant leaves and animal cells, respectively. Localization analysis showed that a portion of N proteins from animal-infecting SBV, SNV and CCHFV colocalized with the PB marker DCP1a-RFP in Vero cells (Figure 1A, white arrow). *In planta*, similarly, the N proteins from plant-infecting counterparts TSWV and RSV were also observed to co-localize with PB marker DCP1-RFP (Figure 1A, white arrow). Earlier studies on the TSWV structural N and glycoproteins showed that these proteins exhibited similar trafficking and localization behavior in animal cells, compared to their structural homologs from animal-infecting bunyaviruses [45,46,47]. Therefore, TSWV N was also expressed in animal cells and its localization relative to PBs analyzed. The results showed that in animal cells, similar to in plant cells, TSWV N protein co-localized with PB (Figure 1A, white arrow). Although earlier studies showed a colocalization of SNV and TSWV N with P bodies, during our studies from all viruses analyzed, irrespective of plant or animal-infecting viruses, the N protein did not completely co-localize with PB upon transient expression. Repeated analyses revealed that a part of the N protein consistently appeared in close proximity to PB. To test whether these signals localized to SG, the cytoplasmic RNA granules that mostly appear adjacent PB, the N proteins from SBV, SNV, CCHFV and TSWV were co-expressed with the SG marker G3BP1 in Vero cells, and subjected to an arsenite treatment for SG induction (Materials and methods). All of the tested N proteins showed partial colocalization with SGs (Figure 1B, white arrow). In plant cells, subjected to a heat treatment for SG induction (Materials and methods), TSWV and RSV N proteins were transiently co-expressed with the SG marker G3BP1-RFP and also showed a colocalization with the SG marker protein to some extent. Similar with the observations made on the PB marker, the TSWV N protein colocalized with the SG marker in both animal and plant cells. 

Aside from the colocalization of N protein to either PB or SG (Figure 1A, white and yellow arrows), the N protein randomly dispersed throughout the cell (Figure 1A, purple arrow). To determine its spatial distribution into more detail, the localization of N relative to the observed PB and SG was quantified (Figure 1C,D). To this end, and to allow statistical analyses, at least 10 cells were collected for each sample/treatment. From all N proteins analyzed, the CCHFV N protein showed the highest degree of co-localization to the observed PB (64%) upon its expression in Vero cells, whereas in 7% of the observed PB, the fluorescence signals of the N protein were localized neighboring PB (Figure 1C). A co-localization of SNV N and PB was observed in only 2% of the observed PB, whereas 24% of the fluorescence signals showed up close to PB. For the SBV N protein, 29% of the observed PB showed a co-localizing N and 12% showed a neighboring localization. Upon expression of the RSV N protein in *N. benthamiana* plant cells, a colocalization with PB was observed in 16% of the cases, whereas 13% localized in close proximity. When TSWV N protein was transiently expressed in Vero cells and *N. benthamiana* cells, the N protein co-localized to PB in 48% and 43% of the observed PB, respectively. A similar variation was observed when the N proteins of SBV, SNV, CCHFV, TSWV and RSV were expressed in Vero cells and/or plant cells, respectively, and the localization was quantified relative to the observed SG (Figure 1D). SBV N showed a high association (78%) with SG in animal cells, whereas RSV N showed a high association (81%) with SG in plant cells. TSWV N protein also colocalized to a high number of SGs in both animal and plant cells. Although the numbers vary in between all viruses, the data altogether indicate that, in contrast to earlier (limited) reports suggesting a co-localization of N with PB only, the N protein from a wide range of viruses of the *Bunyavirales* co-localize with both PB and SG. 

### 3.2. N Protein P Body/SG Association during Viral Infection 

**Figure 2:**
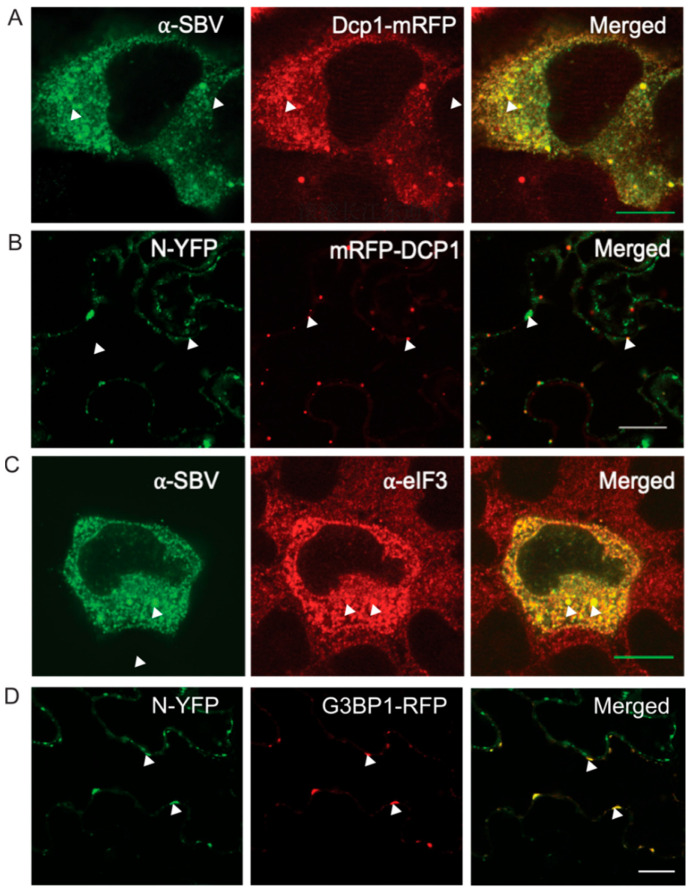
Localization of TSWV-N and SBV-N relative to PB/SG in virus infected cells. (**A**) HsDCP1-mRFP construct was first transfected to the cells and followed by SBV infection. SBV N was detected by immunostaining. (**B**) 5d post TSWV infection, TSWV-N-GFP and AtRFP-DCP1 were agro-infiltrated to infected leaves, confocal microscopy was observed at 48 hpi. (**C**) HsG3BP-mRFP construct was first transfected to the cells and followed with SBV infection. SBV N was detected by immunostaining. (**D**) 5d post TSWV infection, TSWV-N-GFP and AtRFP-G3BP1 were agro-infiltrated to infected leaves, and subjected to heat stress for SG-induction. Confocal fluorescence microscopy was performed at 48 hpi. Scale bar is 10 μm.

To test whether the spatial distribution of transiently expressed N proteins relative to both PB and SG also occurred during virus infection, the experiments were repeated but now in the presence of virus. To this end, TSWV N-GFP and SBV N-GFP were transiently expressed in the additional presence of the PB marker in plant and animal cells, respectively, that were infected with the corresponding viruses prior to the experiment. Thirty hours post transfection with SBV N-GFP and the PB marker, animal cells were infected with SBV. After 24 h, cells were analyzed and showed a strong association of SBV N with P bodies (Figure 2A). Transient expression following TSWV infection was conducted on the infected leaf 5 days post mechanical inoculation. Additionally, here, the results showed a strong association of P bodies with TSWV N (Figure 2B). 

In analogy, transiently expressed N-GFP was localized relative to SGs during a viral infection. In animal cells, the infection with SBV induced formation of SGs (Figure 2C) and a colocalization of N protein with the SG marker was observed (Figure 2C). In planta, TSWV N protein and SG marker were co-expressed in infected leaf tissue 5 days post mechanical inoculation. However, no SG formation was observed in TSWV infected leaf tissue. To stimulate the induction of SGs, plants were subjected to heat stress, after which TSWV N colocalized in most cases of the SG observed (Figure 2D).

### 3.3. TSWV N Protein Preferably Localizes to SG Docked on P-Bodies, and the Formation of SG Docked on PB Is Promoted by Viral Infection

**Figure 3:**
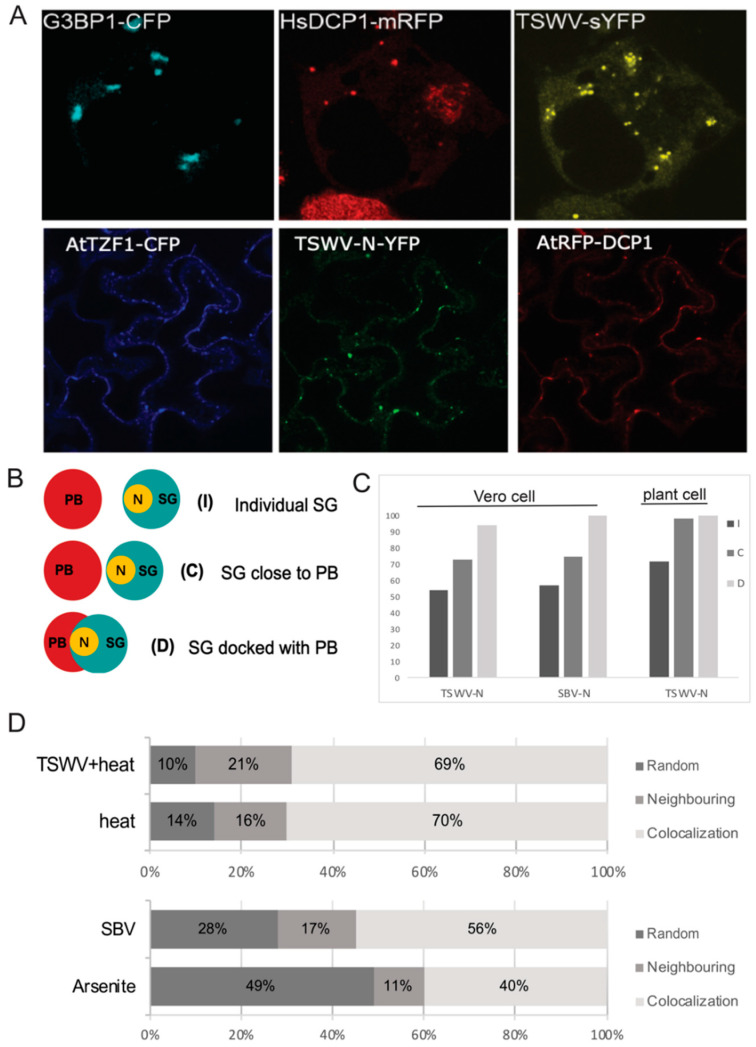
Localization and quantification of N proteins relative to individual PB and SG marker proteins, and PB-SG docking stages. (**A**) Images of TSWV N protein expression in Vero cells, subjected to arsenite treatment for SG-induction, showing the spatial distribution of N protein relative to PB and SG markers (Upper panel). Images of TSWV N protein expression in *N. benthamiana* cells, subjected to a heat shock for SG-induction, showing the spatial distribution of N protein relative to PB and SG markers (lower panel). (**B**) Graphical description of different patterns of N protein associated with SG in different connections to PB (Left panel). The indication “I” represents individual SG distant from PB, “C” represents SG close to PB. “D” represents SG docked on PB. (**C**) Quantification of transiently expressed TSWV and SBV N protein associated with different types of SG, as defined in panel B, in Vero cells, and of TSWV N protein in plant cells. (**D**) Quantification of PB and SG association types in plant cells after TSWV infection + heat shock and after heat shock only (upper half of the panel) and quantification of PB and SG association types in animal cells after SBV infection and after arsenite treatment (lower half of the panel).

Since all N proteins analyzed in this study strongly associated with both cytoplasmic RNA granules when expressed transiently, as well as during a viral infection (for TSWV and SBV), another experiment was performed in which TSWV and SBV N proteins were co-expressed with PB and SG markers simultaneously. In agreement with earlier results, when TSWV N protein was transiently expressed, it localized at SG and at PB, but interestingly also colocalized with SG docked on PB, in both animal cells and plant cells (Figure 3A). In order to quantify the spatial distribution of TSWV N to any of these foci/condensates, PB and SG were first classified into three localization patterns and next the number of cases of TSWV N co-localizing to these stages were counted (Figure 3B). The three defined localization profiles of PB versus SG (Figure 3B) were: individual distribution (I), docking (co-localizing) with SG (D), and neighboring (close) to SG (C). Upon quantification a large number of the docking stage (D) was associated with TSWV N protein, whereas I and C patterns were less enriched in N protein. In Vero cells, 94% of the docking stage D revealed the presence of TSWV N protein and 73% of C pattern was associated with the N protein (Figure 3B). In *N. benthamiana* plant cells, 100% of the PB-SG docking complexes and 98% of closely distributed PB and SG showed the presence of TSWV N protein (Figure 3B). When SBV N protein was transiently expressed in animal cells, 100% of the docking stages D observed showed the presence of SBV N protein, whereas 75% of the neighboring C pattern exhibited an association with N protein (Figure 3B). 

Considering that viral infections are known to influence the occurrence and spatial distribution of cytoplasmic PBs and SGs, experiments were performed to analyze whether viral infections of TSWV and SBV also affected the occurrence and number of SG-PB docking stages. To this end, the numbers of individual PBs and SGs, as well as PB-SG docking stages were counted during either SBV-infection in animal cells or TSWV infection of plant tissues (Figure 3C). In addition, PBs and SGs were also analyzed under non-viral stress conditions for comparison (Figure 3C). In Vero cells, 40% PB docked to SG during arsenite treatment, whereas upon a challenge with SBV, 56% of PB docked to SG (Figure 3C, left panel). Meanwhile, the rate of PB neighboring to SG increased from 11% to 17%. In *N. benthamiana* plant cells, PB docked to SG with a high rate after both heat shock treatment and TSWV infection. When cells were only treated with heat shock, 56% of PB docked to SG. Upon TSWV infection, 63% of PB docked to SG (Figure 3C, right panel). Altogether these data point towards an altering and dynamic distribution profile of N protein relative to the cytoplasmic PBs and SGs, that is not restricted to PB in contrast to earlier reports, and in which a viral infection clearly affects PB and SG dynamics. 

### 3.4. Silencing of Different PB- and SG-Resident Elements Differentially Influences TSWV Replication Rates

**Figure 4:**
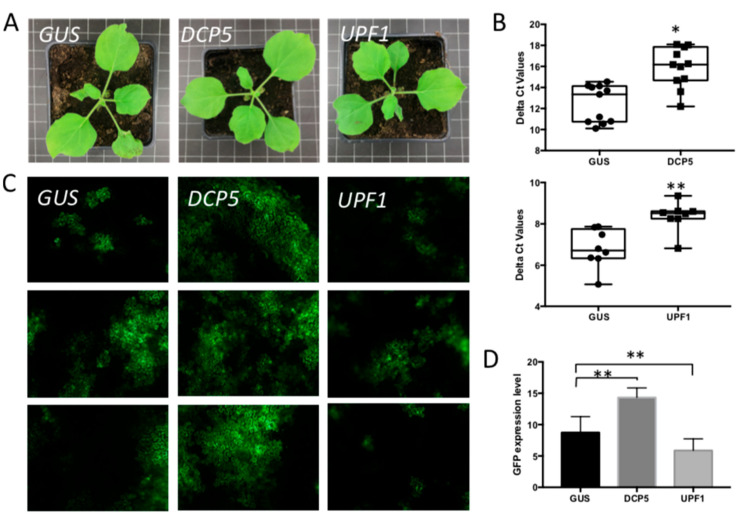
The effect of silencing P body elements on TSWV replication. (**A**) Plant phenotype after VIGS-silencing *DCP5* and *UPF1*, compared with *GUS*-silenced control plant. (**B**) qRT-PCR assay to quantify the silencing efficiency. Non-parametric t test was performed. * *p*-value < 0.05, ** *p*-value < 0.01. (**C**) Green fluorescence resulting from eGFP expression from a TSWV replicon in plants silenced on P body elements, and control plants. Plants were infiltrated TSWV-eGFP replicon constructs 2.5-week post silencing and harvested at around 60 hpi. (**D**) Statistical analysis of GFP intensity in the silenced plants and control plants. A non-parametric t test was performed. * *p*-value < 0.05, ** *p*-value < 0.01.

Considering the clear link to PB and SG, their role as putative sources for capped RNA leader sequences to prime viral genome transcription was hypothesized. In this study, this idea was further investigated using the plant-infecting TSWV as a model system. To this end, Tobacco rattle virus (TRV)-based virus induced gene silencing (VIGS) was performed to first knock down the expression of PB elements and subsequently analyze the effect on viral replication. For PB knock-down, *DCP5* and *UPF1* were selected as two candidate genes. The *DCP5* gene encodes a PB component that coordinates RNA decapping activity with core decapping enzymes [48]. Its silencing will halt decapping and contribute to an increase in PB-resident capped RNA. *UPF1* is a regulator of the nonsense-mediated decay (NMD) pathway and facilitates the transport of nonsense RNA to P bodies for subsequent degradation [49,50]. When plants were silenced on either *DCP5* or *UPF1,* they showed a normal phenotype similar to those from control plants infected by TRV-*GUS* (Figure 4A). When leaf samples were collected and tested by qRT-PCR on *DCP5*/*UPF1* transcriptional expression levels, the delta Ct value indicated that both genes were indeed successfully silenced (Figure 4B). To test the effect on viral replication, these plants were next challenged with TSWV. For easy monitoring and quantification of TSWV replication levels, the recently generated reverse genetics system of TSWV was used [44]. Three weeks post VIGS, plants silenced on *DCP5/UPF1* were infiltrated with TSWV L, M and S-eGFP replicon constructs to rescue TSWV and next analyzed on the relative level of viral replication by quantification of GFP fluorescence resulting from the GFP reporter gene expressed from the S-RNA replicon [44]. To verify the validity of this system, a similar experiment was performed, during which *DCP5*-silenced plants were also challenged with wild-type TSWV virus by mechanical inoculation. Whereas in *UPF1*-silenced plants, TSWV-GFP expression levels were only slightly less (about 30%) than those collected from control plants infected by TRV-*GUS* (Figure 4C,D), in *DCP5*-silenced plants the GFP expression level was higher (about 1.6-fold) than in control plants infected by TRV-*GUS* (Figure 4C). Quantification was performed to show the significance of GFP signals in several silenced plants (Figure 4D). A similar increase in viral replication levels was observed when *DCP5*-silenced plants were challenged with wild-type TSWV virus instead of the TSWV-GFP replicon (Supplemental Figure S1A,B).

**Figure 5:**
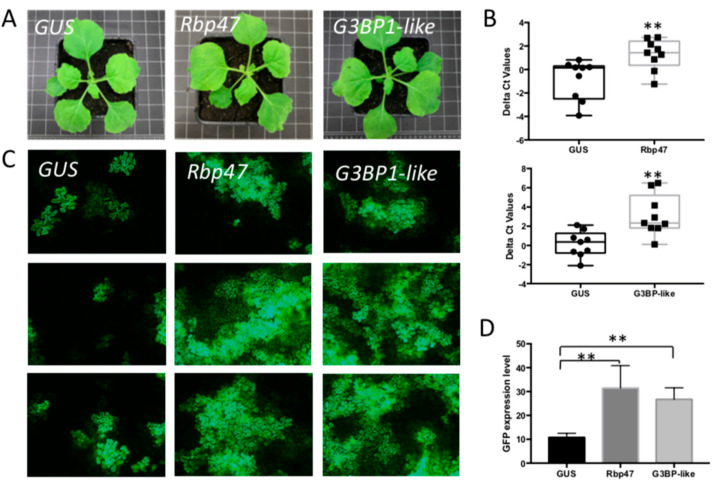
The effect of silencing SG assembly components on TSWV replication. (**A**) Plant phenotype after VIGS-silencing *Rbp47* and *G3BP1-like* genes, compared with *GUS*-silenced control plant. (**B**) qRT-PCR assay to quantify the silencing efficiency. ** *p*-value < 0.01. (**C**) Green fluorescence resulting from eGFP expression from a TSWV replicon in plants silenced on SG assembly components, and control plants. Plants were infiltrated with TSWV-eGFP replicon constructs 2.5-week post silencing and harvested at around 60 hpi. (**D**) Statistical analysis of GFP intensity in the silenced plants and control plants.

In analogy to the knock down of PB elements, TRV-induced gene silencing was also performed on SG components and their effect on TSWV infection analyzed. To this end, several genes were selected. *G3BP1-like* gene and *Rbp47* gene were selected for their function in the assembly of SGs [51]. *EIF4A* and *eIF4E* genes were selected due to the dynamic localization to SGs [51]. When plants were silenced on *G3BP1-like* and *Rbp47* genes, the plant phenotype remained normal (Figure 5A). A qRT-PCR on total RNA purified from silenced leaf samples showed a clear and significant knock down of both genes (Figure 5B, Supplemental Figure S1A). Upon subsequent infiltration of these leaves with TSWV L, M and S-eGFP genome constructs to rescue TSWV and determine the rate of viral replication by GFP fluorescence levels, the expression level of GFP in both *G3BP1-like*- and *Rbp47*-silenced plants increased over two-fold compared with control plants infected by TRV-*GUS* (Figure 5C,D). 

**Figure 6:**
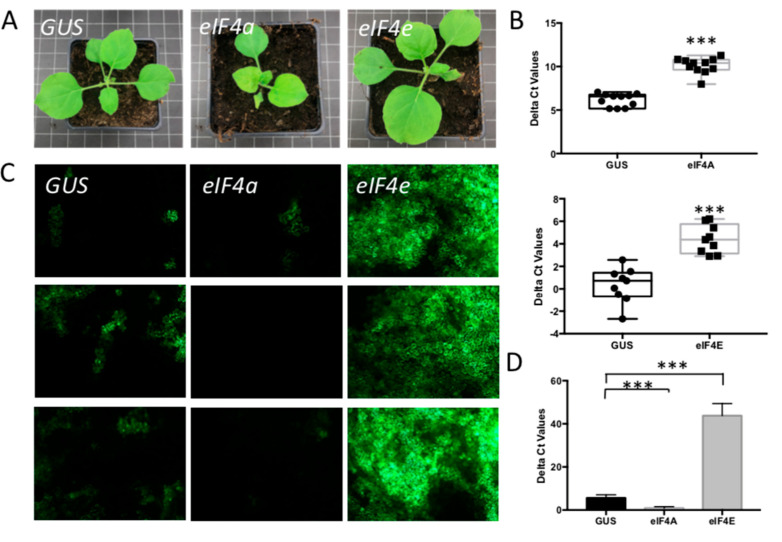
The effect of silencing other SG components on TSWV replication. (**A**) Plant phenotype after VIGS-silencing *eIF4A* and *eIF4E*, compared with the *GUS*-silenced control plant. (**B**) qRT-PCR assay to quantify the silencing efficiency. *** *p*-value < 0.001. (**C**) Green fluorescence resulting from eGFP expression from a TSWV replicon in plants silenced on SG components, and control plants. Plants were infiltrated with TSWV-eGFP replicon constructs 2.5-week post silencing and harvested at around 60 hpi. (**D**) Statistical analysis of GFP intensity in the silenced plants and control plants.

EIF4A and eIF4E are translation initiation factors that enter SGs for storage upon stress induction and are again released to engage in translation of the mRNA when the stress is relieved [51]. Similar to the analysis on *G3BP1-like* and *Rbp47*, plants were silenced on *eIF4A* and *eIF4E* to test the effect on TSWV infection. When the phenotype of silenced plants was checked no difference was observed between *eIF4E*-silenced plants and *GUS*-silenced control plants (Figure 6A). However, plants silenced on eIF4A exhibited stunting and leaf deformation (Figure 6A). A qRT-PCR showed that both genes were effectively silenced (Figure 6B). When those plants were subsequently challenged with a TSWV infection, induced by infiltration of the infectious TSWV L, M and S-eGFP genome constructs, GFP expression level was hardly observed in *eIF4A* -silenced plants compared to *GUS*-silenced control plants. However, and interestingly, *eIF4E*-silenced plants showed an enormously higher GFP expression (about 4 folds than *GUS*-silenced plants), indicating high replication levels of the virus (Figure 6C,D). Additionally, when the experiment with *eIF4E*-silenced plants was repeated but plants this time were challenged with wild-type TSWV virus instead of the TSWV-GFP replicon (Supplemental Figure S1A,B), a similar increase in virus titers was observed. For this reason, hereafter, experiments were performed with the TSWV-GFP replicon system only.

### 3.5. Viral Replication in Plants Simultaneously Silenced on PB-Resident DCP5 and SG-Resident G3BP1-like

**Figure 7:**
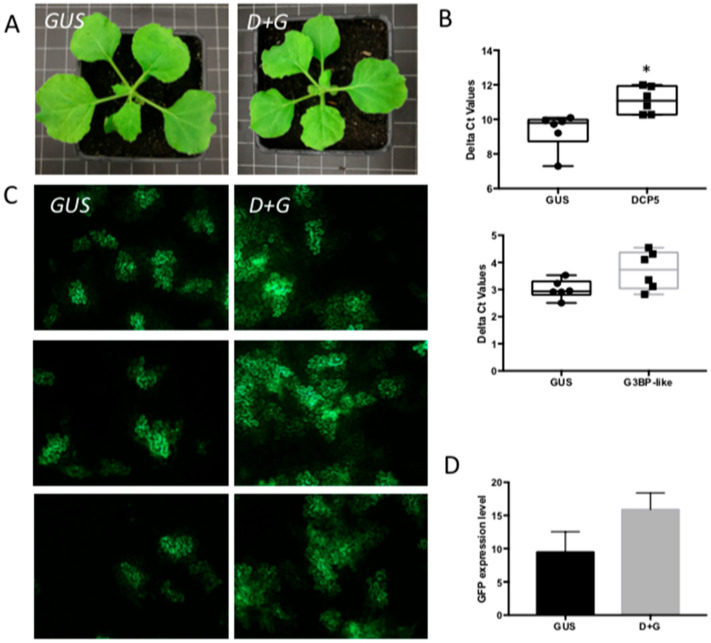
The effect of a simultaneous silencing of *DCP5* and *G3BP1-like* on TSWV replication. (**A**) Plant phenotype after VIGS-silencing *DCP5* and *G3BP1-like*, compared with *GUS*-silenced control plant. (**B**) qRT-PCR assay to quantify the silencing efficiency. * *p*-value < 0.05. (**C**) Green fluorescence resulting from eGFP expression from a TSWV replicon in plants silenced on *DCP5* and *G3BP1-like*, and control plants. Plants were infiltrated with TSWV-eGFP replicon constructs 2.5-week post silencing and harvested at around 60 hpi. (**D**) Statistical analysis of GFP intensity in the silenced plants and control plants.

Considering that the individual silencing of components from PBs or SGs differentially affected viral replication rates, raised the question whether PB and SG would only play a redundant role as a source for capped-RNA leaders, and allowing the virus to alternate between both granules for viral cap snatching. Silencing of one would allow for an escape and promote usage of the other. To test this hypothesis, *N. benthamiana* plants were silenced on *DCP5* and *G3BP1-like* genes simultaneously. Plants did not exhibit a changed phenotype compared to *GUS*-silenced control plants (Figure 7A), and upon qRT-PCR analyses of transcript levels plants were selected that showed silencing of both genes (Figure 7B). When these plants were subsequently infiltrated with TSWV L, M and S-eGFP genome constructs and the rate of viral replication analyzed based on the amount of GFP fluorescence, the level of GFP expression in silenced plants was around 1.5-fold higher than in GUS-silenced control plants (Figure 7C,D). This was somewhat low and unexpected, considering that individual silencing of *DCP5* or *G3BP1-like* earlier revealed a 1.6-and 2-fold increase, respectively, in viral replication, and a stronger cumulative effect was anticipated. 

### 3.6. N Proteins Localize to the Perinuclear Region and Silencing of Nucleocytoplasmic Transport RanGAP Factors Negatively Affects TSWV Replication

**Figure 8:**
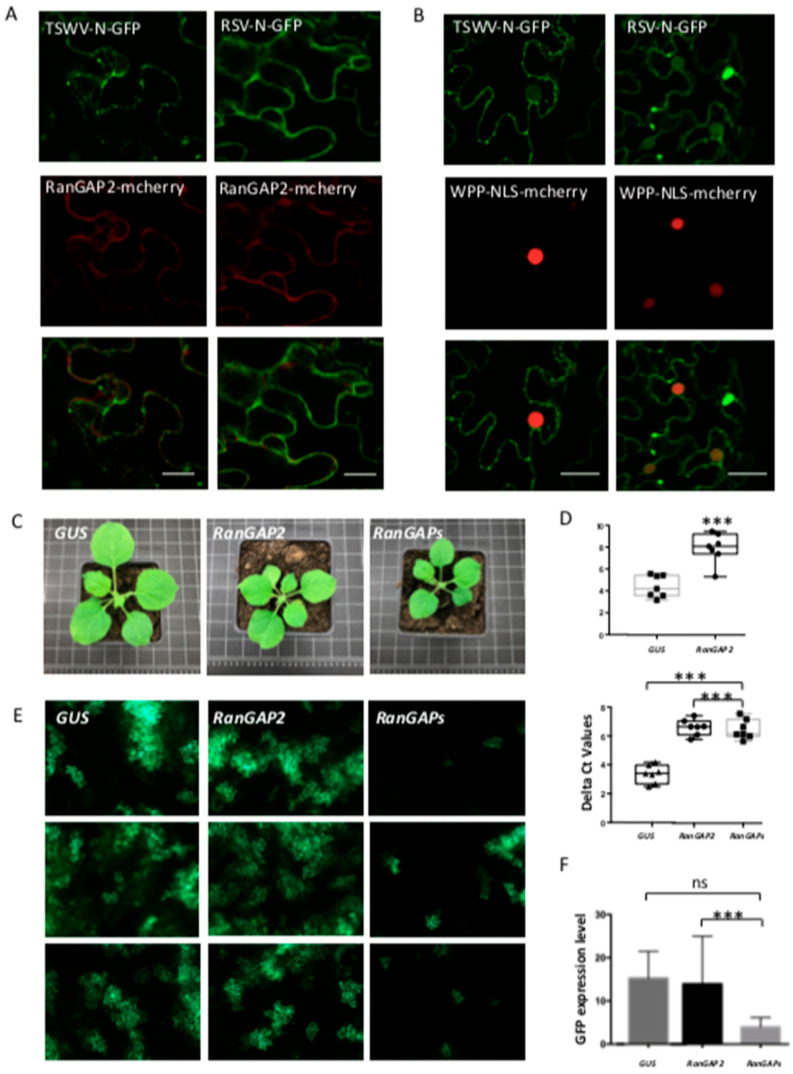
Co-localization analysis of TSWV and RSV N proteins with AtRanGAP2 and the effect of RanGAP silencing on TSWV replication. (**A**) Localization of RSV and TSWV N-GFP protein relative to AtRanGAP2-mcherry in plant cells. Bar presents 10 μm. (**B**) Localization of RSV and TSWV N-GFP protein relative to WPP-NLS-mcherry in plant cells. Bar presents 10 μm. (**C**) Plant phenotype after VIGS-silencing *RanGAPs*, compared with the *GUS*-silenced control plant. (**D**) qRT-PCR assay to quantify the silencing efficiency. *** *p*-value < 0.001. Delta Ct values are shown on Y-axis. (**E**) Green fluorescence resulting from eGFP expression from a TSWV replicon in plants silenced on *RanGAP*(s), and control plants. Plants were infiltrated with TSWV-eGFP replicon constructs 2.5-week post silencing and harvested at around 60 hpi. (**F**) Statistical analysis of eGFP intensity in the silenced plants and control plants.

Since the concomitant silencing of *DCP5* and *G3BP1-like* did not lead to an expected major increase in the viral replication rate resulting from a cumulative effect of their individual silencing, raised the question whether other cytoplasmic foci (upstream of the mRNA trafficking routes leading to SG and PB) could present potential (major) sites/sources for the virus from where to snatch capped-RNA leader sequences as well. Considering that eIF4E, besides being a component of SG, also presents an essential factor for nuclear mRNA export, and its silencing led to a major increase in viral replication (Figure 6B), tempted to hypothesize on the possibility of the nuclear pore complex (NPC) as a potential target/focus for cap snatching. As the gate of nucleocytoplasmic transport, NPCs serve for continuous mRNA export to the cytoplasm and thereby offer a constant efflux/supply of capped RNA. Silencing of *eIF4E* could halt this process, leading to an accumulation of mRNA at NPCs. Prior to testing our hypothesis, the localization of N proteins relative to RanGAP, an important player and marker protein of the nucleocytoplasmic transport machinery at NPCs, was analyzed. Upon *in planta* co-expression of TSWV N-GFP and RSV N-GFP with RanGAP-mcherry, a closer look revealed that both N proteins, besides the observations described above in relation to PB and SG, also revealed a perinuclear localization that overlapped with RanGAP (Figure 8A). When the experiment was repeated, but now in the presence of the RanGAP unique N-terminal domain (WPP domain) fused to a nuclear localization signal (NLS), part of the N protein showed up in the nucleus with RanGAP as well, indicating an interaction of the N protein with the nucleocytoplasmic transport machinery of which RanGAP is a component (Figure 8B). Next, *RanGAP* genes were silenced to inhibit the export and release of nuclear mRNA into the cytosol. Whereas silencing of *RanGAP2* resulted in some slight stunting, silencing of both *RanGAPs* showed a slightly more severe phenotype (Figure 8C). A quantitative RT-PCR was performed to test the silencing efficiency and revealed a significant reduction in the mRNA transcript level (Figure 8D). Subsequently, TSWV L, M and S-eGFP genome constructs were infiltrated to rescue TSWV (containing a S-RNA encoding GFP gene) and determine the rate of viral replication by GFP fluorescence levels. The expression of GFP in single *RanGAP2*-gene-silenced plants was similar to control plants infected by TRV-*GUS* (Figure 8E,F). However, the expression of GFP in plants silenced on both *RanGAPs* simultaneously was clearly lower (~75% reduction) than in control plants infected by TRV-*GUS* (Figure 8E,F). 

### 3.7. TSWV Cleaves Transcripts That End up at Different Locations

**Figure 9:**
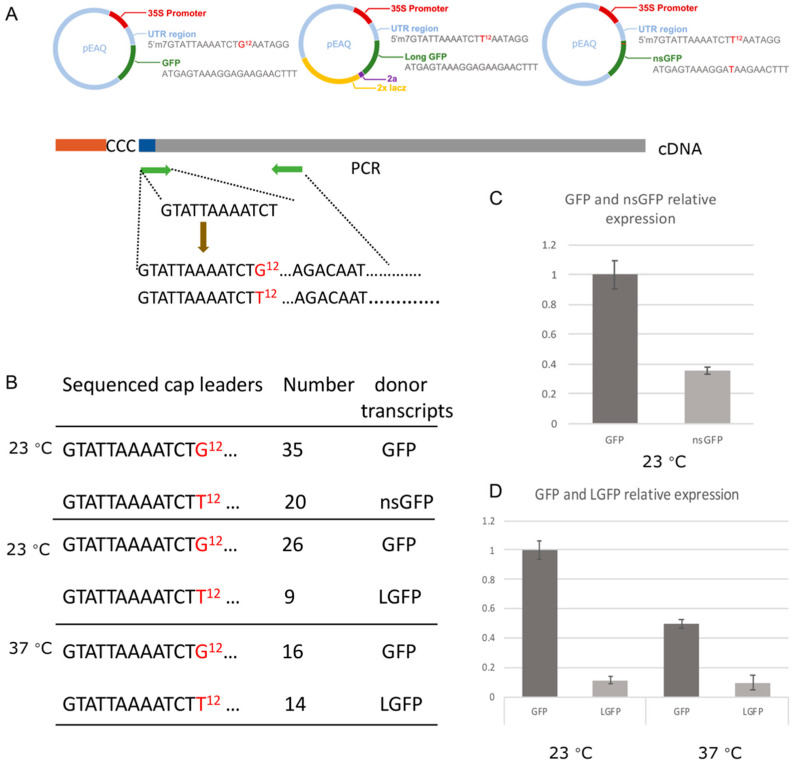
The cloning and identification of capped leader sequences snatched from provided transcripts. (**A**) Schematic overview of GFP, nsGFP and LGFP constructs and the cloning strategy of TSWV mRNA leader sequences. (**B**) The relative distribution of snatched leader sequences collected during in vivo cap donor competition assays between GFP, nsGFP and LGFP transcripts, respectively, at standard condition (23 °C) and heat stress condition (37 °C). (**C**,**D**) Expression levels of GFP, nsGFP and LGFP transcripts as determined by qRT-PCR.

To further analyze the (relative) usage of transcripts from PBs and SGs during TSWV cap snatching/transcription various synthetic transcripts, designed to end up to at PB or SG or reflecting a canonical mRNA, were simultaneously offered to TSWV in plants and their leader sequence usage determined based on sequence analysis of de novo synthesized N mRNAs. To this end, three constructs were made and designed to generate GFP transcripts that ended up at either PB or SG, or engage in normal translation. A first one, named nsGFP, containing a PTC right downstream the start codon of the GFP open reading frame (ORF) and entering the transcripts into the NMD pathway leading to P bodies for further degradation [50] (Figure 9A). A second construct, designated long (L)GFP, containing the GFP ORF fused to a tandem lacZ gene sequence, of which the resulting long transcripts more efficiently stall in translation and store at SGs upon stress induction [52] (Figure 9A). A functional, third, GFP construct, to generate normal translatable transcripts, was used as a control (Figure 9A). To be able to distinguish between leader sequences snatched from either of these transcripts, a marker nucleotide was introduced into each of these at nt position 12 (Figure 9A). 

To test the usage of nsGFP or LGFP transcripts relative to GFP, nsGFP or LGFP constructs were co-infiltrated with GFP to TSWV infected plants in a 1:1 ratio. Experiments were performed in plants kept under normal condition or additionally subjected to a heat treatment (37 °C) for stress induction, to store translationally stalled transcripts into SG. Leaf samples were collected, and total RNA was purified and used for a 5′ race PCR to clone-capped leader sequences from de-novo-synthesized TSWV N mRNA (Figure 9A). PCR fragments obtained, and of the expected size, were purified and ligated into pGEM-T easy vector for sequencing. From the transcript competition assays using GFP and nsGFP, 55 positive clones were collected, of which 35 contained the leader sequence from GFP and 20 from nsGFP, as identified by the marker nucleotide (Figure 9B). From the transcript competition assays using GFP and LGFP, 35 clones were obtained from plants kept at normal condition, of which 26 leader sequences derived from the GFP transcript and 9 from the LGFP transcript. From a similar competition experiment, but during which plants were subject to an additional heat stress, 30 clones were collected of which 16 contained a leader sequence originating from GFP and 14 from LGFP, suggesting an increased usage of the LGFP cap donor under heat stress conditions (Figure 9B).

The expression of all transcripts was verified by qRT-PCR, and revealed that the level of GFP transcripts was about two times higher than that of nsGFP (Figure 9C), and even much higher than LGFP. Heat stress reduced both GFP and LGFP expression (Figure 9D). Despite these differences, all three transcripts were clearly used during TSWV cap snatching with no apparent strong preference for either GFP or nsGFP and heat stress (inducing SG formation and storing long transcripts stalled in translation) affecting the usage of (LGFP) transcripts offered. 

## 4. Discussion

P bodies are currently postulated/considered as the source for cap donors for bunyaviruses, cytoplasmic-replicating segmented NSVs, needed to initiate viral genome transcription [33,53]. In this study, the relation of several bunyaviruses to PBs and other cytoplasmic RNA granules/foci was further investigated. Here, it is shown that the N protein of different plant- and animal-infecting bunyaviruses either transiently expressed or during viral infection, not only localize at PBs but also at SGs (Figure 1) [26,29,54], and preferably at PB-SG docking stages. Silencing of PB and/or SG in plants resulted in differential effects on TSWV titers, but mostly led to higher titers. TSWV/RSV N proteins furthermore revealed a peri-nuclear localization that overlapped with RanGAP2, an important player of the nucleocytoplasmic (RNA) transport pathway through NPCs. Silencing of RanGAPs led to clearly reduced titers of TSWV. These results altogether indicated a possible redundancy of PB and SG in cap snatching and a putative role of sources more upstream in the mRNA trafficking pathway, whose inhibition is more detrimental to the viral genome transcription-replication initiation. 

Data pointing towards PB as source of host cellular mRNAs for cap snatching were first collected from studies performed with the animal-infecting SNV hantavirus. Those studies revealed the usage of capped-RNA leaders from transcripts destined (by the NMD-pathway) to PB for viral genome transcription, and a co-localization of the SNV N protein with PB. Moreover, the SBV N protein exhibited affinity to cap analogue [55], which might explain its localization at foci enriched for mRNA, such as PB. Recently, similar indications have been reported for TSWV N, and the TSWV N protein observed to (partially) localize to PBs as well. However, the usage of capped-RNA leaders from host cellular transcripts destined to PB for TSWV viral genome transcription was not quantified relative to the usage of functional transcripts. Additionally, no other putative sources enriched for host cellular mRNAs were pointed out, nor further investigated whether or not to play a role in the cap snatching process besides PBs, even though SNV was observed to surprisingly still replicate to similar levels in P body deficient cells [53]. The latter findings indicated that the virus likely is able to use other sites/cytoplasmic granules to support viral genome transcription, as also raised by the authors of the study on SNV. Whether these sites are additional or simply just upstream PB, the final destination of any capped-RNA molecule, still remained elusive. The findings from this study indicates at least some redundancy and/or usage of other sources upstream of the cytoplasmic mRNA trafficking pathway. From a rational and deductive point of view, the observed co-localization of N protein to SG is not totally unexpected for several reasons. Firstly, although these granules are only transient and often arise as a result of (a)biotic stress responses, such as viral infections, they contain functional mRNAs that are only stalled in translation. SGs thus provide a perfect substrate for cap snatching, in contrast to PB, where mRNAs are decapped and degraded, and viruses have to protect their mRNAs from becoming cannibalized as cap donor as well. Secondly, with SGs often localizing in close proximity of PB, and even known to exchange components during the formation of docking stages, a co-localization of N proteins to PB tempts to speculate and investigate a co-localization with SG as well. Earlier reports on some bunyaviruses to require translational machinery elements, in particular, active 40S ribosome scanning, to prevent premature transcription termination and support ongoing viral transcription [56,57], support a possible involvement of SGs where such elements (like 40S) can be found. Our results, not only with SNV, for which earlier only a co-localization with PB was reported, but also with several other completely distinct (plant- and animal-infecting) members of the *Bunyavirales*, showing a co-localization of the N protein with PB and SG, strengthened the observations made and support the idea of all these cytoplasmic replicating NSVs performing cap snatching at more foci enriched for host cellular mRNAs, and not limited to PB only.

Previous studies have shown that PB and SG are different granules, but spatially, compositionally, and functionally, they are tightly linked [58]. The formation of PB-SG docking complexes allows for a specific mRNA exchange. Messenger RNAs destined for decay disassemble from polysomes and are first sorted at SG before they subsequently transport into PB [58]. Considering that viral infections often lead to SG formation, viral infections may also boost the RNA interchange between PB and SG. The access of virus to capped RNA from both structures thus would be a very effective way to support viral genome transcription. The observation on both SBV and TSWV N proteins to preferably localize at docked PB/SG supports this point of view.

By taking out DCP5, an enzymatic coactivator of the decapping process in PB [48], more capped-RNA molecules will remain available for cap snatching. As expected, TSWV replication becomes upregulated, as observed in this study, but also observed in another recent study on TSWV and earlier already reported on RVFV where DCP2 silencing in insect cells led to increased viral titers [53]. By taking out UPF1, which interferes at the nonsense-mediated RNA transportation to PB, TSWV replication is only slightly attenuated. A similar result has been shown in mutant Arabidopsis *UPF1*, which reduced the TSWV infection [40]. The slight attenuation in viral replication indicates that NMD RNA-trafficking to PB is probably not the only source of cap donor molecules for TSWV. Notably, a recent study has pointed that *RNA* degradation of molecules entering the NMD pathway seems to occur in “polysomal-derived” complexes, and not at PB [59]. If true, this would further strengthen the possibility of capped RNAs from sources other than/besides PB, e.g., upstream in the cascade of the translational machinery. 

When G3BP1-like or Rbp47, both components from SGs, another putative cap donor source, were taken out the viral titer of TSWV in plants turned much higher. In other words, breaking SG assembly promoted viral replication and indicated antiviral activity of SGs, as already observed and reported with many viruses. However, and alternatively, SGs may also act redundantly with PB. When the virus accesses PB more efficiently and silencing of SGs shifts some of its stalled mRNA to PB, viral genome transcription would benefit, and titers go up. However, SGs are not always present in the cell, not even and always during viral infections, but when they are formed during biotic/abiotic stress conditions and translatable mRNAs are stalled/stored, they present an additional/alternative escape for cytoplasmic replicating segmented NSVs to support cap snatching. It thus makes sense that when cells are inhibited in SG formation, these viruses still maintain their ability to replicate well. To further confirm if SGs indeed facilitate cap snatching, experiments are needed to analyze the usage of cap leader sequences that originate from transcripts transported to SGs. Furthermore, it remains to be investigated whether the silencing of SG formation does lead to a shift in the stalled mRNAs to PBs. 

In the case that PB and SG present the sole source for capped-RNA leaders to support cap snatching, a combined silencing of DCP5 (leading to increased levels of capped RNA in PB) and G3BP (leading to a possible shift of stalled mRNA to PB) would lead to a major increase in viral transcription-replication levels. Unexpectedly, and surprising, only a slight increase in TSWV replication was observed, and not up to a higher level than when these granules were taken out individually. This raises the question on the use of additional/alternative sources/sites from where to take capped RNA, more upstream of the RNA trafficking pathway leading to PB/SG. Earlier studies on SNV already showed that even when cells were depleted from PB the virus was still able to effectively replicate. When summarizing the observations on PB and SG, it remains to be noted that these condensates not only reflect end points of enriched RNAs but are also relatively easy to discern. This might blur our views and lead to ignoring possible upstream sources of first-hand capped RNA molecules for viral cap snatching. This idea is also supported by the observation that TSWV does not seem to exhibit a strong preference for either translationally functional, SG-directed or NMD-PB-directed mRNAs during in vivo transcription competition assays. The application of heat stress also did not drastically change their usage, and even functional transcripts were still being used. 

**Figure 10:**
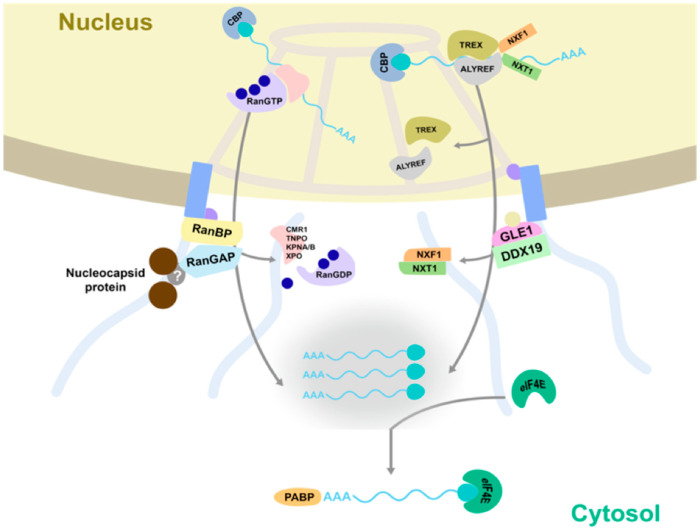
A model for cap snatching at the nucleopore. The interaction of TSWV nucleocapsid (N) protein with RanGAP is indicated. N protein localizes at the nucleopore through interaction with RanGAP and thereby acquires access to the capped RNA molecules involved in RanGAP/RanBP-mediated transportation, and to the mRNA pool (resulting from GLE/DDX19-mediated transportation) in the exclusion zone of the nuclear pore complex at which cap binding protein exchanges with eIF4E, and continues trafficking to the cytosol.

The localization of TSWV and RSV N protein in the perinuclear region, partly overlapping with RanGAP, an important player of the nucleocytoplasmic transport machinery at the NPC, pointed towards the idea of accessing mRNA during nucleocytoplasmic transport through nuclear pore complexes (Figure 10). A perinuclear localization of N protein is not entirely novel since this has earlier been reported for TSWV and Capsicum chlorosis virus (CaCV) [47,60], but also for bunyaviral N and ribonucleoprotein (RNP) complexes [33,53,60,61,62,63,64]. As the gate of nucleocytoplasmic transport, NPCs serve for continuous mRNA export to the cytoplasm and thereby offer a constant efflux/supply of capped RNA. Several papers have indicated the exclusion zone at NPC to allow the exchange of certain proteins and RNAs, and in which the movement of cargos is regulated by glycine- leucine-phenylalanine-glycine (GLFG) domains at the cytoplasmic side [65,66]. Intriguingly, TSWV N localization altered by a RanGAP domain fused with nuclear localization signal, and suggests an interaction between N and RanGAP. Silencing of both *RanGAP* (1 and 2) genes strongly affected TSWV replication and indicates that inhibition of the nuclear-cytoplasmic mRNA trafficking pathway is quite detrimental to viral replication. RanGAP interacts with RanBP2 and this latter binds to the nuclear pore filaments that face the cytoplasm. Its silencing could thus cause a reduction in the pool of mRNAs at the cytoplasmic side of NPC, resulting from a reduced RanGAP-mediated mRNA nuclear efflux release [67] (Figure 10). Concomitantly, its silencing/absence at NPC could prevent access of TSWV N to the nuclear efflux of capped RNA (Figure 10). This idea agrees with data obtained on *eIF4E* silencing, where an increase in TSWV replication was observed. Knowing that eIF4E is also involved in nuclear export of specific mRNAs, in specific on the pick-up of mRNAs from the nuclear efflux at NPC, for further release in the cytoplasm, the hypothesis on nuclear pore as first-hand source has become quite appealing (Figure 10). Furthermore, in addition to the localization at SG during stress conditions, G3BP1 is an RNA-binding protein which interacts with nucleoporin filaments at the cytoplasmic side of NPC as well [68]. Both G3BP1 and eIF4E silencing could thus lead to the retaining of mRNAs at the NPC exclusion zone, due to their delivery to the cytosol being halted. Whether G3BP1 and eIF4E silencing indeed leads to an accumulation of capped RNA accumulation at the (cytoplasmic side of) NPC remains to be analyzed. Considering that eIF4E is also involved in other processes, such as protecting mRNA from degradation and initiating translation, as well as being resident in SGs, underlines the importance of more studies to be performed on this point. In light of all this, it is interesting to note that for the Tacaribe and Junin Arenaviruses replication-transcription complexes (RTC) are shown to contain translation initiation factors and G3BP and localize to cytosolic puncta containing viral N protein [69]. However, these RTCs do not associate with specific SG and PB markers such as Dcp1a and Xrn1, respectively, or TIA1, PABP or HSP70, nor co-localize with Golgi proteins, whereas they do contain phosphatidylinositide PI4P, a protein that is typically found on the cytoplasmic side of Golgi membranes. The latter suggests that intact Golgi do not appear to present the sites of viral replication-transcription. Whether arenavirus RTCs reside in vicinity of the perinuclear Golgi and nuclear envelop/NPC from where they could directly access (via G3BP and/or RanGAP?) a source of capped RNA remains speculative. 

Although initial studies with SNV indicated PB as the source for supply of capped RNA leader sequences to support cap snatching, it is clear that the cytoplasmic replicating bunyaviruses exhibit a more complex cross talk to cytoplasmic condensates that is not limited to PB, as observed in this study. This idea is also strengthened by the fact that many key regulators of cytoplasmic mRNA translation and decay also include nucleocytoplasmic shuttling (e.g., Pab1, Xrn1, eIF4G, and eIF4E) and are found to dictate nuclear events such as transcription, splicing, and export of certain mRNAs [70,71,72]. The explanation on their silencing and subsequent effects on viral genome transcription initiation may thus not be as simple and strictly correlated to one foci/RNA granule as the current dogma, in which bunyaviruses only use P bodies as a source for capped RNA leaders to initiate genome transcription. 

How and where cap snatching occurs still is not clear, but one hypothesis could be that snatching of capped-RNA leader sequences from cytoplasmic host mRNAs involves a targeting of mRNAs via these so-called “coordinators”, proteins that are involved in the functionally coupling of nuclear transcription and cytoplasmic mRNA stability, translation and decay [71].

Findings on the rate of TSWV replication upon silencing of various elements from PB, SG, and also NPC, have provided support for the idea that these viruses might access other (upstream) sources enriched in mRNA. Future experiments using an animal-infecting bunyavirus such as SBV, in analogy to those performed with TSWV in plants in this study, are needed to confirm and strengthen this hypothesis.

## Data Availability

Data will be available from the corresponding author at reasonable request.

## Supplementary Materials

The following supporting information can be downloaded at: https://www.mdpi.com/article/10.3390/v14081679/s1, Figure S1. The effect of silencing *DCP5* and *eIF4E* on viral replication. Table S1. Primers used in this study.
