# Supplementary material for: Bunyaviral N Proteins Localize at RNA Processing Bodies and Stress Granules: The Enigma of Cytoplasmic Sources of Capped RNA for Cap Snatching
Source: Viruses. 2022 Jul 29;14(8):1679. doi: 10.3390/v14081679 (PMC9414089; doi:10.3390/v14081679)
Supplement: Supplementary file 1 [file viruses-14-01679-s001.zip › Supplemental Figure 1.pdf]

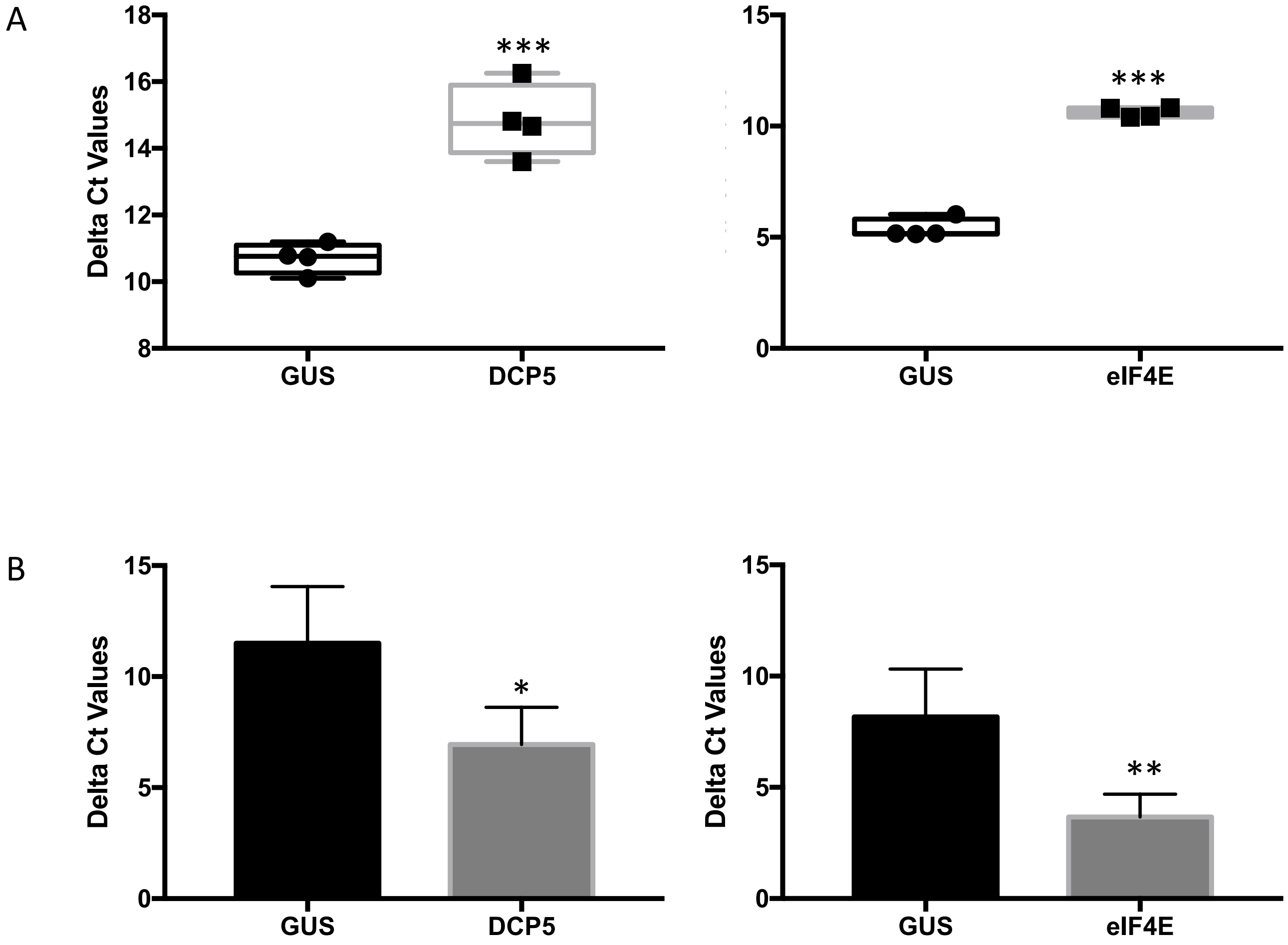

Supplemental Figure S1. The effect of silencing of DCP5 and eIF4E on viral replication. (**A**) qRT-PCR assay to quantify the silencing efficiency. * p-value < 0.05， ** p-value < 0.01, *** p-value < 0.001. (**B**) TSWV titer represented by N gene expression value on silenced plants and control plants. * p-value < 0.05， ** p-value < 0.01, *** p-value < 0.001
