# Supplementary material for: Bunyaviral N Proteins Localize at RNA Processing Bodies and Stress Granules: The Enigma of Cytoplasmic Sources of Capped RNA for Cap Snatching
Source: Viruses. 2022 Jul 29;14(8):1679. doi: 10.3390/v14081679 (PMC9414089; doi:10.3390/v14081679)
Supplement: Supplementary file 1 [file viruses-14-01679-s001.zip › Supplemental table 1.pdf]

Table S1: Primers used in this study.

Name	Sequence
F_SacII_eGFP	AACCGCGGATGGTGAGCAAGGGCGAG
R_SacII_eGFP	AACCGCGGTTACTTGTACAGCTCGTC
F_SacII_mRFP	ACCGCGGATGGCCTCCTCCGAGGAC
R_SacII_mRFP_stop	ACCGCGGTTAGGCGCCGGTGGA
F_attB1_N_TSWV	GGGGACAAGTTTGTACAAAAAAGCAGGCTTAACCATGTCTAAGGTTAAGCTCACT
R_attB2_N_TSWV	GGGGACCACTTTGTACAAGAAAGCTGGGTAAGCAAGTTCTGCGAGTTT
F_attB1_N_SBV	GGGGACAAGTTTGTACAAAAAAGCAGGCTTAACCATGTCAAGCCAATTCATTTTT
R_attB2_N_SBV	GGGGACCACTTTGTACAAGAAAGCTGGGTAGATGTTGATACCGAATTG
F_attB1_N_SNV	GGGGACAAGTTTGTACAAAAAAGCAGGCTTAACCATGAGCACCCTCAAAGAAGTG
R_attB2_N_SNV	GGGGACCACTTTGTACAAGAAAGCTGGGTAAAGTTTAAGTGGTTCTTG
F_attB1_adapt	GGGGACAAGTTTGTACAAAAAAGCAGGC
R_attB2_adapt	GGGGACCACTTTGTACAAGAAAGCTGGGT
F_attB1_CCHFV_N	CAAAAAAGCAGGCTCCACCATGGAAAACAAAATCGAAG
R_attB2_CCHFV_N	CAAGAAAGCTGGGTAGCGCCAACATCATCT
F_attb1_G3BP1	GGGGACAAGTTTGTACAAAAAAGCAGGCTCCACCATGGTGATGGAGAAGC
R_attb2_G3BP1	GGGGACCACTTTGTACAAGAAAGCTGGGTACTGCCGTGGCGCAAGC
F_attB1_caprin1	GGGGACAAGTTTGTACAAAAAAGCAGGCTCCACCATGCCCTCGGCCACC
R_attB1_caprin1	GGGGACCACTTTGTACAAGAAAGCTGGGTAATTCACTTGCTGAGTGTTCAT
F_attB1_DCP1A	GGGGACAAGTTTGTACAAAAAAGCAGGCTCCACCATGGAGGCGCTGAGT
R_attB2_DCP1A	GGGGACCACTTTGTACAAGAAAGCTGGGTATAGGTTGTGGTTGTCTTTG
F_q_PCR_G3BP	AGTAGAGGAACCTGAAGAAAGACAGC
R_q_PCR_G3BP	CCATGTCATTACTGACAACTGCCTG
F_q_PCR_GAPDH	CACCAGGGCTGCTTTTAACTC
R_q_PCR_GAPDH	GGAATTTGCCATGGGTGGAATC
F_q_PCR_SBV	TCAGATTGTCATGCCCCTTGC
R_q_PCR_SBV	TTCGGCCCCAGGTGCAAATC
F_DCP1a_qPCR	TATCACCAGCATCGCAGACC
R_DCP1a_qPCR	TGGTAAGGGGAAGCTGACCT
F_attB1_N_EMARaV	GGGGACAAGTTTGTACAAAAAAGCTGAACAGATGCCTATTATTCCAAAGCC
R_attB2_N_EMARaV	GGGGACCACTTTGTACAAGAAAGCTGAACTTGTGGTTGTTTTGAAGCTTTC
F_attB1_N_RSV	GGGGACAAGTTTGTACAAAAAAGCTGAACAGATGGGTACCAACAAGCCAG
R_attB2_N_RSV	GGGGACCACTTTGTACAAGAAAGCTGAACTCTAGTCATCTGCACCTTCTGC
F_RNAi_Nb-eIF4E-like	GGGGACAAGTTTGTACAAAAAAGCTGAACAGGGAAAGTGGACAATGAGCTTTTC
R_RNAi_Nb-eIF4E-like	GGGGACCACTTTGTACAAGAAAGCTGAACTCTACGCAGAATAACGATTCTTGG
F_BglII_AteiF4E	GAAGATCTATGGCGGTAGAAGACACTCC
R_BglII_AteiF4E	GAAGATCTTCAAGCGGTGTAAGCGTTC
F_BglII_AtTZF1	GAAGATCTATGATCGGCGAAAATAAAAACC
R_BglII_AtTZF1	GAAGATCTACCGAGTGAGTTCTCTCTACTGAG
F_RNAi_Nb-DCP5	GGGGACAAGTTTGTACAAAAAAGCTGAACAGAATCTGTCAAGATCATCTCTC
R_RNAi_Nb-DCP5	GGGGACCACTTTGTACAAGAAAGCTGAACTTCTGTGTGTGTGGCCTTTAC
F_q_PCR_TSWV	GCTTCCCACCCTTTGATT
R_q_PCR_TSWV	TCCCGAGGTCCTTGTATT
F_q_PCR_eif4E-like	GTGAGGTGGAAGAGGAAGG
R_q_PCR_eif4E-like	GGAGAAGGTGTAAATGGGTC
F_q_PCR_DCP5	AATAACGGTGTCTGCTCTG
R_q_PCR_DCP5	CAGTTTGCGAGATTGTGG
F_q_PCR_actin	TCCTGATGGGCAAGTGATTAC
R_q_PCR_actin	TTGTATGTGGTCTCGTGGATTC
F_q_PCR_EF1a	AGCTTTACCTCCCAAGTCATC
R_q_PCR_EF1a	AGAACGCCTGTCAATCTTGG
RBP47_qPCR_F	GGTCTCCGTGAAAATACCTGC
RBP47_qPCR_R	GAAAGACGAACTGCCTGCTT
RBP47_RNAi_F	GGGGACAAGTTTGTACAAAAAAGCAGGCTTATAGCACTGGTGAAAAGCGTG
RBP47_RNAi_R	GGGGACCACTTTGTACAAGAAAGCTGGGTATTGTTGCTGTGCTGATGGTT
G3BP_RNAi_F	GGGGACAAGTTTGTACAAAAAAGCAGGCTTAATGATGCAGTCCAGGTTCCA
G3BP_RNAi_R	GGGGACCACTTTGTACAAGAAAGCTGGGTACTAGAAGGTGCTGGAGGAGG
G3BP_qPCR_F	TGTGGTCGAGGAGAAGAGGT
G3BP_qPCR_R	TAACCCCTGCCACCTCCATA
Nbeif4a-RNAi-F	GGGGACAAGTTTGTACAAAAAAGCAGGCTTAcaactttctgctctggagt
Nbeif4a-RNAi-R	GGGGACCACTTTGTACAAGAAAGCTGGGTAtccttgaaacctcttgagagc
UPF1_RNAi-F	GGGGACAAGTTTGTACAAAAAAGCAGGCTTAATTGCGTTATTCAGGGGATGC
UPF1_RNAi-R	GGGGACCACTTTGTACAAGAAAGCTGGGTACCAAAACGGCGAGGAAGTGTA
